# The Cognitive Connectome in Healthy Aging

**DOI:** 10.3389/fnagi.2021.694254

**Published:** 2021-08-18

**Authors:** Eloy Garcia-Cabello, Lissett Gonzalez-Burgos, Joana B. Pereira, Juan Andres Hernández-Cabrera, Eric Westman, Giovanni Volpe, José Barroso, Daniel Ferreira

**Affiliations:** ^1^Department of Clinical Psychology, Psychobiology and Methodology, Faculty of Psychology, University of La Laguna, La Laguna, Spain; ^2^Division of Clinical Geriatrics, Center for Alzheimer Research, Department of Neurobiology, Care Sciences, and Society, Karolinska Institutet, Stockholm, Sweden; ^3^Clinical Memory Research Unit, Department of Clinical Sciences, Lund University, Lund, Sweden; ^4^Department of Neuroimaging, Centre for Neuroimaging Sciences, Institute of Psychiatry, Psychology and Neuroscience, King’s College London, London, United Kingdom; ^5^Department of Physics, University of Gothenburg, Gothenburg, Sweden; ^6^Department of Radiology, Mayo Clinic, Rochester, MN, United States

**Keywords:** connectome, cognition, aging, graph theory, compensation, differentiation

## Abstract

**Objectives**: Cognitive aging has been extensively investigated using both univariate and multivariate analyses. Sophisticated multivariate approaches such as graph theory could potentially capture unknown complex associations between multiple cognitive variables. The aim of this study was to assess whether cognition is organized into a structure that could be called the “cognitive connectome,” and whether such connectome differs between age groups.

**Methods**: A total of 334 cognitively unimpaired individuals were stratified into early-middle-age (37–50 years, *n* = 110), late-middle-age (51–64 years, *n* = 106), and elderly (65–78 years, *n* = 118) groups. We built cognitive networks from 47 cognitive variables for each age group using graph theory and compared the groups using different global and nodal graph measures.

**Results**: We identified a cognitive connectome characterized by five modules: verbal memory, visual memory—visuospatial abilities, procedural memory, executive—premotor functions, and processing speed. The elderly group showed reduced transitivity and average strength as well as increased global efficiency compared with the early-middle-age group. The late-middle-age group showed reduced global and local efficiency and modularity compared with the early-middle-age group. Nodal analyses showed the important role of executive functions and processing speed in explaining the differences between age groups.

**Conclusions**: We identified a cognitive connectome that is rather stable during aging in cognitively healthy individuals, with the observed differences highlighting the important role of executive functions and processing speed. We translated the connectome concept from the neuroimaging field to cognitive data, demonstrating its potential to advance our understanding of the complexity of cognitive aging.

## Introduction

Cognitive aging has been extensively investigated. A common approach has been to focus on how a particular cognitive function changes over time or differs across age groups, using univariate methods for data analysis (West, 2001; Tisserand and Jolles, 2003; Lachman, 2004; Schroeder and Salthouse, 2004; Schaie, 2005; Salthouse, 2009, 2010, 2016; Harada et al., 2013; Ferreira et al., 2015; Reas et al., 2017; Oschwald et al., 2019). While this approach has provided important insight on age-related cognitive decline, cognitive functions are highly interrelated with each other through complex associations, possibly in a dynamic manner across ages, i.e., these inter-relations may differ across age groups. Capturing such complex associations is difficult when focussing on a particular cognitive function in isolation, as conventionally done with univariate analysis. This motivated the development and use of multivariate approaches, facilitating a deeper and more integrated understanding of cognitive aging (Salthouse and Ferrer-Caja, 2003; Viroli, 2012; Hoogendam et al., 2014; Habeck et al., 2015; Nielsen and Wilms, 2015; Salthouse et al., 2015; Machado et al., 2018). Previous multivariate studies have informed on the complex association between cognitive performance and key demographic, clinical, and neuroimaging variables. However, it is still largely unknown how cognitive domains and cognitive components are organized and interrelated with each other, forming a structure that could be called the “cognitive connectome.” Further, whether this cognitive connectome changes during aging has not been investigated so far. Within the connectome field of neuroscience (Bullmore and Sporns, 2021), graph theory has recently emerged as a promising technique to investigate complex associations in the data, both in normal and pathological aging.

Graph theory enables the analysis of complex inter-relationships between multiple measures. Although graph theory has been extensively applied to neuroimaging data in the field of aging (Zhu et al., 2012; Jung et al., 2018; Chong et al., 2019; Lee et al., 2019; Xia et al., 2019), to our knowledge only one study has applied graph theory on cognitive data in an aging study (Gonzalez-Burgos et al., 2020). In that study, Gonzalez-Burgos et al. (2020) studied compensation of age-related differences in verbal fluency and demonstrated the potential of graph theory to investigate cognitive aging, as an alternative to other multivariate methods such as random forest analysis or orthogonal partial least squares to latent structures (Machado et al., 2018; Gonzalez-Burgos et al., 2020). To our knowledge, four other previous studies applied graph theory on cognitive data, in other fields than normal aging: three studies investigated children with epilepsy (Garcia-Ramos et al., 2015, 2016; Kellermann et al., 2015), and another study investigated neurological patients with different etiologies (Jonker et al., 2019). Hence, translating the concept of connectome from the neuroimaging field to cognitive data (i.e., the “cognitive connectome”) is timely and is expected to provide relevant new insights on how human cognition is organized. This step is warranted in order to understand the behavioral outcomes of the well-studied brain connectome in neuroimaging research (Van den Heuvel and Sporns, 2019; Bullmore and Sporns, 2021). This understanding could have various implications, both for research and clinical work in normal and pathological aging.

The overall goal of the current study was to investigate cognitive aging with graph theory using a large set of cognitive measures (47 variables) in three groups of age spanning from 37 to 78 years (early-middle-age = 37–50 years; late-middle-age = 51–64 years, and elderly = 65–78 years). The first aim was to investigate how multiple cognitive domains and cognitive components are interrelated with each other in the whole cohort, forming a cognitive connectome independent of age. To address this first aim, we applied modular analyses using graph theory. The second aim was to investigate whether this cognitive connectome differs between groups of early-middle-age, late-middle-age, and elderly individuals. To address this second aim, we also applied modular analyses using graph theory, across the three groups of age. The third aim was to gain a deeper understanding of network features underlying age-related differences in the cognitive connectome. To address this third aim, we quantified and analyzed global and nodal measures from the three age groups. Based on previous studies from our group (Ferreira et al., 2015; Machado et al., 2018) and other groups (Lachman, 2004; Willis et al., 2010), we hypothesized that differences between the two middle-age groups would be modest, but the differences would be more prominent when comparing middle-age groups with the elderly group. We anticipated more disconnected cognitive networks in the elderly group, with an important role of executive functions and processing speed in network differences, as predicted by the executive and processing speed theories of cognitive aging (Salthouse, 1996; West, 1996).

## Materials and Methods

### Participants

A total of 334 participants were selected from the GENIC (Group of Neuropsychological Studies of the Canary Islands) database (Ferreira et al., 2017). All individuals were native Spanish speakers from the Canary Islands, with ages between 37 and 78 years and a balanced distribution of sex across age. For the current study, participants were selected according to the following criteria: (1) No dementia according to a Mini-Mental State Examination (MMSE) score ≥24, a Blessed Dementia Rating Scale (BDRS) score <4, and a Functional Activities Questionnaire (FAQ) score <6; (2) No mild cognitive impairment based on consensus diagnosis from two experienced neuropsychologists, following Winblad et al. (2004) criteria applied on comprehensive neuropsychological assessment (Ferreira et al., 2015) and age-, sex-, and education-corrected normative data; (3) Right-handed manual preference as assessed by the Edinburgh Handedness Inventory. We applied this criterion because some cognitive functions such as language abilities (Springer et al., 1999), visuospatial functions (Zaidel, 1990; Kong et al., 2018), or attention (Heilman, 1995) are lateralized and so, the “cognitive connectome” could be different in left-handed individuals; (4) No abnormal findings such as stroke, tumors, or hippocampal sclerosis on MRI according to an experienced neuroradiologist; and (5) No neurologic or psychiatric disorders, systemic diseases with neuropsychological consequences, or history of substance abuse. An exception was made for the BDRS. Although the BDRS scale cut-off for abnormality is frequently established at ≥4 points (Blessed et al., 1968; Erkinjuntti et al., 1988), the “changes in personality, interests and drive” subscale may influence the BDRS total score and does not necessarily reflect functional impairment. With the aim of excluding only individuals with functional impairment, we included those participants with total BDRS scores ≥4 (*n* = 15) if: (a) 70% or higher percentage of the BDRS total score resulted from the “changes in personality, interests and drive” subscale; and (b) if a score ≤1.5 was obtained in the other two subscales (“changes in performance of everyday activities” and “changes in habits”). The same procedure has been applied in previous studies (Cedres et al., 2019; Gonzalez-Burgos et al., 2020). Participation was completely voluntary and all subjects gave written informed consent in accordance with the Declaration of Helsinki. The study was approved by the local ethics committee of the University of La Laguna (Spain).

### Cognitive Assessment

A comprehensive neuropsychological protocol was applied covering the following cognitive domains: processing speed, attention, executive functions, premotor functions, episodic memory, procedural memory, visuoconstructive, visuoperceptive, and visuospatial functions, and language. The protocol is fully detailed in Supplementary Table 1 and described elsewhere (Ferreira et al., 2015). In addition, the MMSE (Folstein et al., 1975) was used as a measure of global cognition, and the BDRS (Blessed et al., 1968) and the FAQ (Pfeffer et al., 1982) were used as measures of functional status. The Wechsler Adult Intelligence Scale (WAIS-III) Information subtest (Wechsler, 1997a) was scored and used as an indicator of crystallized intelligence.

### Graph Analysis

All cognitive variables detailed in Table 1A were selected as the nodes to construct the network constituting the cognitive connectome.

**Table 1:** Cognitive variables and cognitive modules.

(A) Cognitive variables included as the nodes in graph analysis		(B) Cognitive modules from modular analysis with the Newman algorithm
LM A-Immediate	TAVEC 1st trial	**Verbal Memory Module**
LM B1-Immediate	TAVEC Learning	
LM B2-Immediate	TAVEC Interference	
LM A-Delay	TAVEC Short delay	
LM B-Delay	TAVEC Short delay-Clues	
LM A-Recognition	TAVEC Long delay	
LM B- Recognition	TAVEC Long delay-Clues	
VR I-Total Score	8/30 Long delay	**Visual Memory and Visuospatial Module**
VR II-Total Score	FRT	
VR-Copying	JLOT-First half	
VR- Total Recognition	JLOT-Second half	
8/30 1st trial	BNT	
8/30 Learning	Spatial Span backward	
8/30 Interference	Block Design Total	
8/30 Short delay		
STROOP Words	Digit Span forward	**Executive Functions and Premotor Functions Module**
STROOP Colors	Digit Span backward	
STROOP Inhibition	Spatial Span forward	
Phonemic fluency	Luria’s HAM Right	
Semantic fluency	Luria’s HAM Left	
Action fluency	Luria’s—Coordination	
PCV Decision time	CTT-Part 1	**Processing Speed Module**
PCV Motor time		
HT 1st trial	HT Long delay	**Procedural Memory Module**
HT Learning		

*The table shows the cognitive variables used as the nodes in the network construction (A). The table also shows the cognitive variables included in each of the different cognitive modules (B). Please see Supplementary Table 1 for more information about cognitive variables and tests. LM, Logical Memory; FRT, Facial Recognition Test; JLOT, Judgment of Line Orientation Test; VR, Visual Reproduction; BNT, Boston Naming Test; HT, Hanoi Tower; PCV, PC-Vienna System; CTT, Color Trails Test*.

Regarding our first aim of investigating an age-independent cognitive connectome in the whole cohort, all cognitive variables were corrected for age and crystallized intelligence (WAIS-III Information subtest) by using multiple linear regression prior to network construction. The resulting residual values were used for network construction. We controlled the age because we aimed to investigate an age-independent cognitive connectome, and we also controlled for crystallized intelligence because it is known to have a strong impact on cognitive performance (Ferreira et al., 2016).

Regarding our second aim of investigating whether the cognitive connectome differs between age groups of early-middle-age, late-middle-age, and elderly individuals, new cognitive networks were built separately for each age group by controlling only for the effect of crystallized intelligence, using multiple linear regression prior to network construction. Again, the resulting residual values were used for network construction.

The edges between the nodes were calculated through matrices of Pearson correlation coefficients from each pair of nodes. Matrices were binarized by thresholding the correlation coefficients at a range of densities from 15% to 50% of connections, in steps of 1%. This ensured the exclusion of disconnected networks (densities below 15%) and random topologies (densities above 50%, when the small-world index became close to 1). Network topologies were compared across this range of densities. Results from global graph measures were reported across all densities. Results from nodal graph measures were considered also across all densities but reported only at the median density (30%), to simplify reporting and as a common procedure to represent the whole range of densities (Pereira et al., 2018; Ferreira et al., 2019). Both self-connections and negative correlations were excluded.

Regarding our third aim of gaining a deeper understanding of the network features underlying potential age-related differences in the cognitive connectome, the nodal and global graph measures described below were calculated. Some graph measures have been reported to be unstable, especially in small cohorts (Mårtensson et al., 2018). To circumvent this issue, we aimed for age groups larger than those in studies shown to be reproducible (Welton et al., 2015), and selected graph measures that are stable according to Mårtensson et al. (2018).

**Figure 1:**
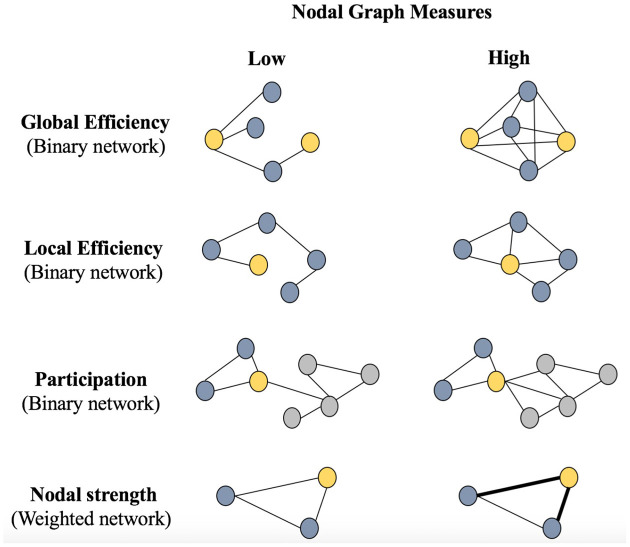
Schematic representation of nodal graph measures. Circles represent nodes and edges represent connections between the nodes. The Global Efficiency panel represents a less efficient connectivity between the two yellow nodes (left: two steps between yellow nodes) vs. a more efficient connectivity between the two yellow nodes (right: multiple connections of one and two steps between yellow nodes). The Local Efficiency panel represents a less efficient connectivity between the yellow node and all other nodes (left: only one connection) vs. a more efficient connectivity between the yellow node and all other nodes (right: four connections). The Participation panel represents two communities (blue and gray); the yellow node belongs to the blue community and has less connections (edges) with the gray community in the left part of the panel (low participation) than in the right part of the panel (high participation). The nodal strength panel represents the intensity of the connectivity of the yellow node with its connections, where thinner connections (left) represent a lower strength and thicker connections (right) represent a higher strength. Parentheses indicate whether binary or weighted networks were used for each nodal measure.

Figure 1 shows a graphical representation of the nodal graph measures included in this study. The nodal *global efficiency*, the *local efficiency*, and the *participation* coefficient, which were calculated from the binary networks across the different densities. In addition, we also included the *nodal strength*, which was calculated from the weighted network (before binarization). The nodal *global efficiency* is the average of the inverse shortest path length from a node to all other nodes in the network. The *local efficiency* is the global efficiency of a node calculated on the subgraph created by the node’s neighbors. The *participation* coefficient quantifies the relation between the number of edges connecting a node outside its community and its total number of edges. The *nodal strength* is the sum of the weights of all edges connected to a node.

**Figure 2:**
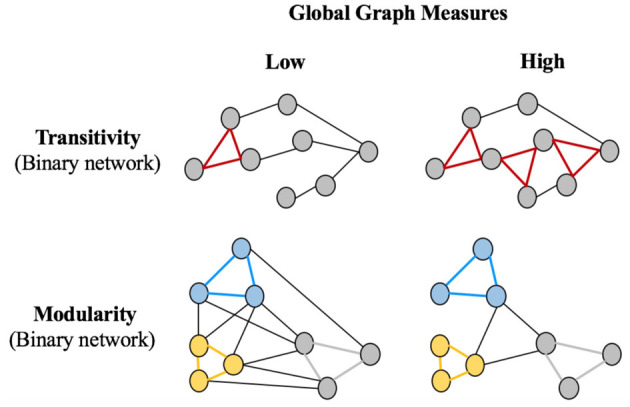
Schematic representation of global graph measures. Circles represent nodes and edges represent connections between the nodes. The Transitivity is the number of triangles in a network, with the left panel showing a lower transitivity (one triangle), vs. the right panel showing a higher transitivity (three triangles). The Modularity panel shows that there are three modules (blue, yellow and gray), which can be less clearly divided into separate communities on the left part (high between-module connectivity relative to within-module connectivity: low modularity) than on the right part (low between-module connectivity relative to within-module connectivity: high modularity). Parentheses indicate that binary networks were used for the Transitivity and Modularity global measures. The global measures of Global Efficiency, Local Efficiency and Average Strength were also used in this study but are not represented in this Figure because they are similar to the representation in Figure 1, but for the global network instead of the local network.

Figure 2 shows a graphical representation of the global graph measures included in this study: the *average global efficiency*, the *average local efficiency*, the *transitivity*, and the *modularity*. All these measures were calculated in the binary networks across the different densities. In addition, we also included the *average strength*, which was calculated from the weighted network (before binarization). Global measures such as the *average global efficiency*, the *average local efficiency*, and the *average strength* represent the mean of all nodes across the whole network for each nodal measure. The *transitivity* refers to the fraction of a node’s neighbors that are also neighbors of each other in the whole network, normalized by the whole network. Hence, the *transitivity* reflects how well the nodes are connected to nearby nodes forming cliques. The *modularity* is a quantitative measure that reflects the extent to which a graph can be divided into clearly separate communities (that is, subgraphs or modules). In addition, complementary modular analyses were performed using the Newman algorithm (Newman, 2004) to provide qualitative information on how cognitive variables are organized into specific communities.

### Statistical Analysis

To address the aims of the current study, we stratified individuals into three age groups by dividing the whole age range from 37 to 78 years into equidistant groups with an age range of 13 years each: an early-middle-age group (37–50 years) treated as the reference group, a late-middle-age group (51–64 years), and an elderly group (65–78 years). We favored three age groups because three is the smallest number of groups to test for non-linear group differences. In particular, we used ANCOVA (see below) to test both linear and non-linear trends in cognitive performance across age groups. Also, we ensured that the resulting groups had sizes larger than previous studies shown to be reproducible (Welton et al., 2015). We further confirmed that the sizes of the age groups provided enough power for the comparisons using ANCOVA (see below).

Because graph analysis is based on correlations, cognitive variables that showed low variability were excluded from the analysis. More specifically, among all variables included in our comprehensive neuropsychological protocol (Ferreira et al., 2015), we excluded error variables, the PASAT (Gronwall, 1977), recognition variables in all our memory tests, and the discrimination test from Visual Reproduction (Wechsler, 1997b). This gave a total of 47 cognitive variables included in our graph analysis (Table 1A). We ensured that the selection of 47 cognitive variables covered all cognitive domains and their subcomponents. Further, missing data could not be accommodated in our software, and given the multivariate nature of graph theory, individuals with at least one missing value in any of the included 47 cognitive variables were excluded from this study. We used the *BRAPH software version 1.0.0* (Mijalkov et al., 2017) for graph analysis and *R Studio version 0.99.483* with the *ULLRToolbox* for statistical analyses.

The widely used Newman algorithm (Newman, 2004) was implemented for modular analyses, to reduce the set of 47 cognitive variables down to a few modules (Table 1B). This analysis also served as our computational method to identify cognitive connectomes. Since different algorithms can produce different modular solutions and thus affect the identification of the cognitive connectome, we replicated all our analyses with another widely used method: the Louvain algorithm (Blondel et al., 2008). Pearson’s correlation was used to test the relationship between educative level and crystallized intelligence (WAIS-III Information subtest). ANCOVA and Chi square tests were used for continuous and categorical variables, respectively. A *p*-value <0.05 was deemed significant in all analyses. In addition, the false discovery rate (FDR) adjustment was used at *p* ≤ 0.05 (two-tailed) for analyses involving nodal graph measures (Genovese et al., 2002).

## Results

### Key Characteristics of the Whole Cohort and the Three Age Groups

**Figure 3:**
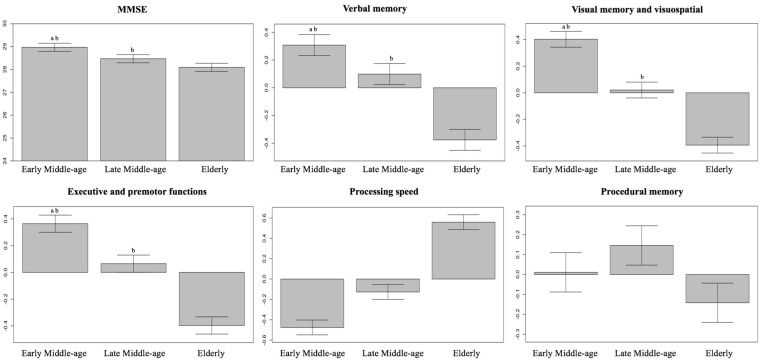
Differences in cognitive performance across age groups. Between-group differences in MMSE and cognitive modules were analyzed using ANCOVA with crystalized intelligence (WAIS-III Information subtest) as a covariate. Processing speed is an inverse measure, so that a higher score denotes a worse performance. Whiskers show 95% confidence intervals. ^a^Significantly different from the late-middle-age group. ^b^Significantly different from the elderly group. MMSE, Mini-Mental State Examination.

Table 2 shows the key characteristics of the whole cohort (*N* = 334) and the three age groups. Briefly, there were no group differences in sex distribution, but educative level and scores in crystallized intelligence (WAIS-III Information subtest) differed across ages. Hence, we controlled for the effects of the educative level/WAIS-III Information subtest when investigating cognitive performance across age groups. Since there was a strong correlation between the educative level and WAIS-III Information subtest (*r* = 0.7, *p* < 0.001), we favored the continuous nature of WAIS-III Information subtest to be used as a covariate in further analyses. In order to characterize cognitive profile across age groups, the set of 47 cognitive variables were reduced into five cognitive domains or modules using modular analysis with the Newman algorithm. After controlling for the WAIS-III Information subtest in ANCOVA, we observed lower MMSE scores and lower performance in all the five cognitive modules with increasing age (Table 2, Figure 3). More specifically, we observed a significant linear trend in executive and premotor functions, verbal memory, visual memory, and visuospatial measures. This means that the magnitude of the difference between the early-middle-age group and the late-middle-age group was similar to the magnitude of the difference between the late-middle-age group and the elderly group. In contrast, we observed a significant quadratic trend in procedural memory and processing speed, indicating comparable performance between the two middle-age groups, and significantly worse performance in the elderly group. All tests with ANCOVA showed power values of 1 or close to 1, indicating good statistical power.

**Table 2:** Key characteristics of the whole cohort and the three age groups.

	Whole cohort (*N* = 334)	Early-middle-age (*n* = 110)	Late-middle-age (*n* = 106)	Elderly (*n* = 118)	
	M(SD)/count(%)	M(SD)/count(%)	M(SD)/count(%)	M(SD)/count(%)	*p*-value
Age (years) (min-max)	57.85 (11.2) (37–78)	44.6 (3.4)^a,b^ (37–50)	57.8 (4.4)^b^ (51–64)	70.2 (3.8) (65–78)	<0.001
Sex (female, count (%))	188 (56.0%)	58 (52.7%)	63 (59.4%)	67 (56.8%)	<0.61
Education level					
Illiteracy	4	0	0	4	<0.001
Unfinished primary studies	43	2	6	35	
Completed primary studies	120	48	31	41	
Completed secondary studies	73	34	20	19	
University studies	94	26	49	19	
WAIS-III Information	15.3 (6.3)	15.7 (5.8)^a,b^	17.6 (6.4)^b^	12.9 (5.9)	<0.001
MMSE	28.5 (1.5)	29 (1.3)^a,b^	28.6 (1.4)^b^	27.9 (1.6)	<0.001
Verbal memory module	0 (1)	0.3 (0.6)^a,b^	0.2 (0.7)^b^	−0.5 (0.6)	<0.001
Visual memory and visuospatial module	0 (1)	0.4 (0.4)^a,b^	0.1 (0.5)^b^	−0.5 (0.6)	<0.001
Executive and premotor functions module	0 (1)	0.4 (0.5)^a,b^	0.2 (0.7)^b^	−0.6 (0.6)	<0.001
Processing speed module	0 (1)	−0.5 (0.4)^a,b^	−0.2 (0.5)^b^	0.7 (0.8)	<0.001
Procedural memory module	0 (1)	0.0 (0.7)	0.1 (0.8)^b^	−0.1 (0.7)	<0.05

*The table shows mean (SD) except for sex and education level, where count (%) is shown. The five cognitive modules were obtained using the Newman algorithm in the whole cohort when reducing the set of 47 cognitive variables into modules. All 47 cognitive variables had previously been corrected for age and crystallized intelligence (WAIS-III Information subtest). The three age groups were then compared across modules to investigate age-related differences in cognitive performance. ANCOVA with crystallized intelligence (WAIS-III Information subtest) as a covariate was used to test between-group differences in MMSE and cognitive modules. Processing speed is an inverse measure so that a higher score denotes a worse performance. ^a^Significantly different from the Late-middle-age group. ^b^Significantly different from the Elderly group. MMSE, Mini-Mental State Examination. WAIS, Wechsler Adult Intelligence Scale*.

### Aim 1—Age-Independent Cognitive Connectome

**Figure 4:**
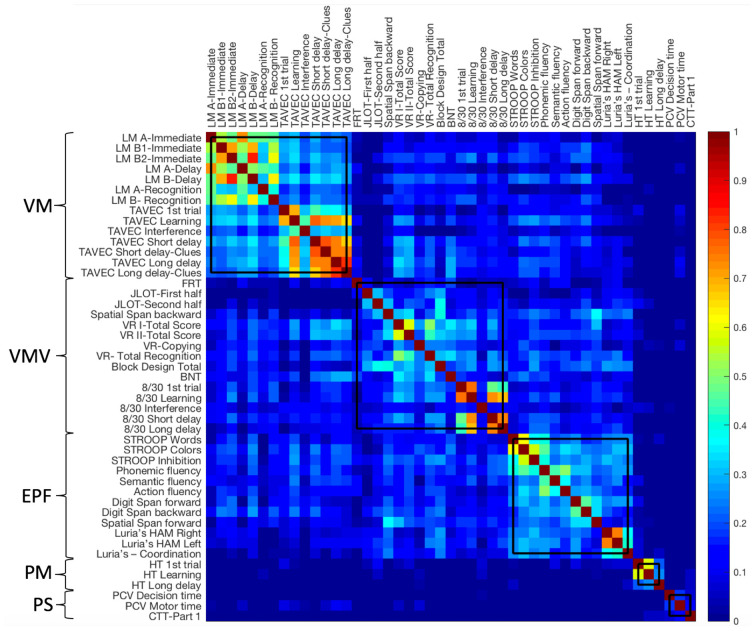
Age-independent cognitive connectome in the whole cohort. Weighted correlation matrix in the whole cohort (*N* = 334) sorted out by cognitive modules obtained with the Newman algorithm. Pearson’s correlation coefficients were used to build the matrix. The color bar indicates the strength of the Pearson’s correlation coefficients: colder colors represent weaker correlations, while warmer colors represent stronger correlations. PM, procedural Memory module; VM, verbal memory module; PS, processing speed module; VMV, visual memory and visuospatial abilities module; EPF, executive functions and premotor functions module; LM, logical memory; FRT, facial recognition test; JLOT, judgment of line orientation test; VR, visual reproduction; BNT, boston naming test; HT, hanoi tower; PCV, PC-Vienna System; CTT, color trails test.

To investigate how cognitive domains and cognitive components are organized and interrelated with each other (i.e., to determine the “cognitive connectome”), independently of age, we corrected our 47 cognitive variables by de-trending the effects of age and WAIS-III Information subtest. The age-independent cognitive connectome was then investigated through a modular analysis (Newman algorithm) performed on the 47 de-trended cognitive variables. As it can be seen in Figure 4, memory variables of verbal nature clustered together forming a first module. Memory variables of visual nature clustered together and with visuospatial measures, forming a second module. Executive and premotor functions also clustered together, forming the third module. Variables of procedural memory and processing speed formed the fourth and fifth small separate modules, respectively. Between-module correlations were observed between the modules of verbal memory, visual memory—visuospatial measures, and executive—premotor measures. However, procedural memory and processing speed modules showed weak almost non-existent between-module correlations. The Louvain algorithm showed the same results except for procedural memory and processing speed modules converging into one single module (Supplementary Figure 1).

### Aim 2—Age-Related Differences in the Cognitive Connectome

We then investigated whether the cognitive connectome differs across age groups of early-middle-age, late-middle-age, and elderly individuals. This time, performance in our 47 cognitive variables were corrected only for WAIS-III Information, by de-trending its effect.

**Figure 5:**
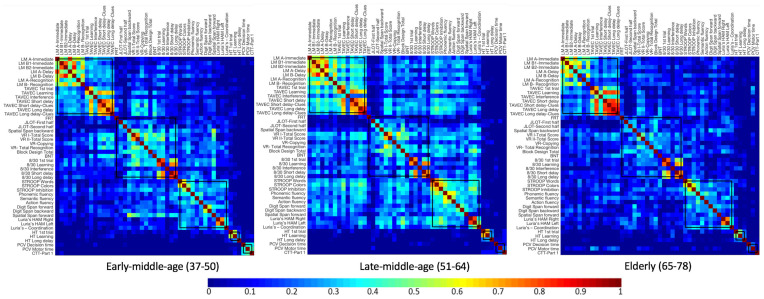
Correlation matrices for each age group, keeping the five modules from the whole cohort fixed. Newman algorithm was used for modular analysis. Pearson’s correlation coefficients were used to build the matrix. The color bar indicates the strength of the Pearson’s correlation coefficients: colder colors represent weaker correlations, while warmer colors represent stronger correlations. See Supplementary Figures 2–4 for matrices with larger size and labelled regions. LM, Logical Memory; FRT, Facial Recognition Test; JLOT, Judgment of Line Orientation Test; VR, Visual Reproduction; BNT, Boston Naming Test; HT, Hanoi Tower; PCV, PC-Vienna System; CTT, Color Trails Test.

Firstly, we visually inspected the five modules from the age-independent cognitive connectome fixed across the three age groups, in order to describe the correlation matrices used for the quantitative analyses of global and nodal measures described below (Figure 5, see Supplementary Figures 2–4 for matrices with larger size). The correlation matrix in the early-middle-age group was very similar to that of the age-independent cognitive connectome. Nonetheless, in the early-middle-age group, the visual memory—visuospatial module showed stronger between-module correlations with executive—premotor and verbal memory modules. Likewise, the correlation matrix of the late-middle-age group was similar to that of the early-middle-age group, although with a tendency to show weaker within-module correlations in the visual memory—visuospatial module, stronger within-module correlations in verbal memory and executive—premotor modules, and stronger between-module correlations of the executive—premotor module with verbal memory and visual memory—visuospatial modules. The elderly group showed overall weaker correlations than the other two age groups. However, the elderly group was the only group where processing speed variables showed correlations with cognitive variables from other modules.

**Figure 6:**
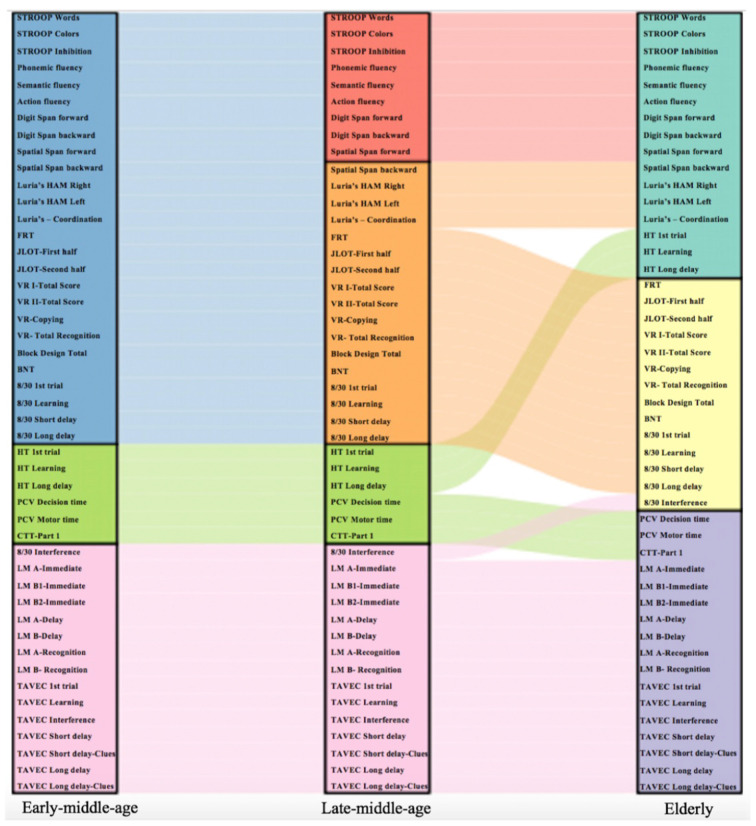
Cognitive variables included in each cognitive module for each age group. The alluvial plot shows how each cognitive variable flows across the cognitive modules in each age group. The Newman algorithm was used for modular analysis. Each cognitive module is represented with a different color: blue color represents an executive and premotor, visual memory and visuospatial functions module; green color represents a procedural memory and processing speed module; pink color represents a verbal memory module; red color represents an executive functions module; orange color represents a premotor functions, visual memory and visuospatial functions module; turquoise color represents a procedural memory, executive and premotor functions module; yellow color represents a visual memory and visuospatial functions module; and violet color represents a verbal memory and processing speed module. Schematic representation of cognitive modules using Newman and Louvain algorithms are detailed in Supplementary Figures 5, 6, respectively.

Secondly, in order to quantify the differences described on visual inspection of correlation matrices, we conducted new modular analyses (Newman algorithm), separately within each age group. These modular analyses tested whether different cognitive modules emerge in each age group. Our results showed rather similar cognitive connectomes across the three age groups. The most notable differences were observed in visual and executive domains, which emerged as only one module in the early-middle-age group, but split into two separate modules in late-middle-age and elderly groups (Figure 6). Likewise, while verbal memory and processing speed were separate modules in early-middle-age and late-middle-age groups, the two modules converged into one single module in the elderly group (Figure 6). The Louvain algorithm showed very similar results. The only exception was that in the elderly group, the procedural memory module converged with the visual memory—visuospatial module instead of converging with the executive—premotor module (Supplementary Figure 6).

### Aim 3—Global and Nodal Network Differences in the Cognitive Connectome Across Age Groups

**Figure 7:**
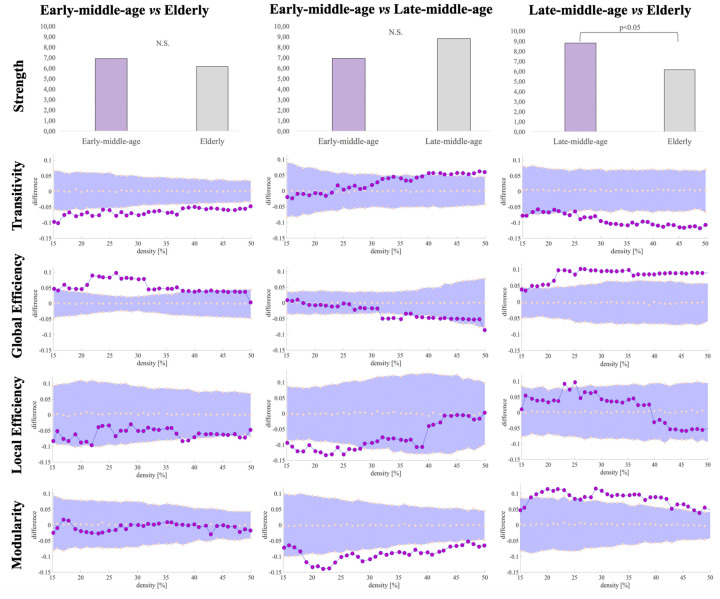
Global network differences in the cognitive connectome across age groups. Figures illustrating modularity, local efficiency, global efficiency and transitivity measures display network densities on the x-axis, spanning from min = 15% to max = 50%, in steps of 1%. Between-group differences in network measures are displayed on the y-axis. Between-group differences are significant when the red circles fall out of the purple-shaded area. N.S., non-significant (*P* > 0.05).

We compared global and nodal network measures calculated from correlation matrices of early-middle-age, late-middle-age, and elderly groups. Significant densities are detailed between brackets in the following sentences. Regarding global network measures, the elderly group showed decreased transitivity in all densities (15%–50%) and increased average global efficiency measures (17%–39%) when compared with the reference early-middle-age group (Table 3, Figure 7). The late-middle-age group showed decreased average global efficiency (32%–36%; 38%–43%), average local efficiency (16%–18%; 20%–25%), and modularity measures (19%–24%; 28%–47%) when compared with the reference early-middle-age group (Table 3, Figure 7). The comparison between the elderly group and the late-middle-age group showed that the elderly group had increased average global efficiency (19%–50%) and modularity measures (19%–24%; 29%–43%) and decreased average strength and transitivity measures (26%–50%; Table 3, Figure 7).

**Table 3:** Differences in global network measures from the cognitive connectome across age groups.

	Early-middle-age vs. Elderly	Early-middle-age vs. Late-middle-age	Late-middle-age vs. Elderly
Av. Strength	n.s	n.s	↓
Transitivity	↓	n.s	↓
Global Efficiency	↑	↓	↑
Local Efficiency	n.s	↓	n.s
Modularity	n.s	↓	↑

*The table shows an overview of the results from global graph analyses. Please, see Figure 7 for more details about quantitative results. Each column shows the between-group differences in global network measures, with the first group as the reference group in the comparison: higher values in the second group in the comparison are indicated with an upwards arrow (e.g., in the “Early-middle-age vs. Elderly” comparison, an upwards arrow indicates that the Elderly group had a higher global efficiency than the Early-middle-age group), lower values in the second group in the comparison are indicated with a downwards arrow, and n.s denotes non-significant results. Results were considered significant when p ≤ 0.05 (two-tailed)*.

Regarding nodal network measures, the elderly group showed a decrease in local efficiency and nodal strength in processing speed variables and an increase in global efficiency in procedural memory and processing speed variables, when compared with the reference early-middle-age group (Table 4). Further, the elderly group showed an increase in the participation coefficient in executive and visuospatial variables, compared with the reference early-middle-age group. The late-middle-age group showed a decrease in local efficiency and an increase in the participation coefficient in processing speed when compared with the reference early-middle-age group. Finally, the comparison between elderly and late-middle-age groups showed that the elderly group had an increase in global efficiency in processing speed and visuoperceptive variables and an increase in the strength in processing speed variables. Further, the elderly group showed a decrease in local efficiency in executive variables, when compared with the late-middle-age group.

**Table 4:** Differences in nodal network measures from the cognitive connectome across age groups.

		Early-middle-age vs. Elderly	Early-middle-age vs. Late-middle-age	Late-middle-age vs. Elderly
**Global Efficiency**	PCV Motor time	↑(0.006)	n.s	↑(<0.001)
	PCV Decision time	↑(0.006)	n.s	↑(<0.001)
	CTT-Part 1	↓(0.006)	n.s	n.s
	HT 1st trial	↑(0.006)	n.s	n.s
	HT Learning	↑(0.006)	n.s	n.s
	HT Long delay	↑(0.006)	n.s	n.s
	FRT	n.s	n.s	↑(<0.001)
**Local Efficiency**	PCV Motor time	n.s	↓(<0.001)	n.s
	PCV Decision time	↓(<0.001)	↓(<0.001)	n.s
	HT Long delay	↑(<0.001)	n.s	↑(<0.001)
	CTT-Part 1	↓(<0.001)	↓(<0.001)	n.s
	STROOP Words	↓(<0.001)	n.s	n.s
	STROOP Inhibition	n.s	n.s	↓(<0.001)
	Semantic fluency	n.s	n.s	↓(<0.001)
**Participation**	STROOP Words	n.s	↑(0.003)	n.s
	STROOP Colors	↑(0.006)	↑(0.003)	n.s
	STROOP Inhibition	↑(0.006)	n.s	n.s
	Block Design Total	↑(0.006)	n.s	n.s
**Nodal Strength**	PCV Motor time	↑(<0.001)	n.s	↑(<0.001)
	PCV Decision time	↓(<0.001)	n.s	n.s
	CTT-Part 1	↓(<0.001)	n.s	n.s

*Each column shows between-group differences in local network measures, with the first group as the reference group in the comparison: higher values in the second group in the comparison are indicated with an upwards arrow (e.g., in the “Early-middle-age vs. Elderly” comparison, an upwards arrow indicates that PCV motor time shows a higher global efficiency in the Elderly group than the Early-middle-age group), lower values in the second group in the comparison are indicated with a downwards arrow, and n.s denotes non-significant results. False discovery rate (FDR) adjustment was used at *p* ≤ 0.05 (two-tailed) for analyses. FDR adjusted *p*-value within parentheses. PCV, PC-Vienna System; CTT, Color Trails Test; HT, Hanoi Tower; FRT, Facial Recognition Test*.

## Discussion

We investigated how multiple cognitive domains and cognitive components are interrelated with each other, forming a cognitive connectome. We also investigated whether this cognitive connectome and its network characteristics differ between early-middle-age, late-middle-age, and elderly groups. Our results showed a cognitive connectome that illustrates the organization of cognitive functions and cognitive components into five modules. This cognitive connectome was rather stable across age groups but some differences were observed in the modular organization, the strength of correlations between cognitive variables, and the characteristics of the networks. Our results support the relevance of executive functions and processing speed in cognitive aging, and stress the potential of graph theory to unravel the complex organization of cognition.

Our first aim was to investigate how multiple cognitive domains and cognitive components are interrelated with each other, forming a cognitive connectome. We wanted to identify a cognitive connectome that is independent of age and other relevant confounding factors such as crystallized intelligence. To that end, we regressed out the effect of age and crystallized intelligence previous to our modular analysis. We identified a cognitive connectome that included five modules: verbal memory, visual memory—visuospatial functions, procedural memory, processing speed, and executive—premotor functions. These modules fit with the cognitive structure reported in the study by Salthouse and Ferrer-Caja (2003), which included “space” abilities (similar to our visual memory—visuospatial module), reasoning (similar to our executive module), verbal memory, and processing speed. Despite the extensive literature on cognitive aging, we are not aware of studies that had an explicit focus on the complex inter-relations between cognitive domains and cognitive components, apart from the study of Salthouse and Ferrer-Caja (2003). However, several studies that applied analytical methods for the reduction of cognitive data are of interest in this discussion. Using different approaches such as factorial analysis (Mitchell et al., 2012; Viroli, 2012; Hayden et al., 2014; Mungas et al., 2014; Nielsen and Wilms, 2015; Salthouse et al., 2015; Rizio and Diaz, 2016) and principal component analysis (Oh et al., 2012; Costa et al., 2013; Aribisala et al., 2014; Fellows and Schmitter-Edgecombe, 2015), four factors (i.e., modules) form the most frequently reported cognitive structure across all these studies. Among these four factors, verbal memory, executive function, and processing speed are the most common factors across studies. The fact that a rather stable solution of modules has repeatedly been reported irrespectively of the cohort, age range, cognitive tests used, and analytical method employed, highlights the potential existence of a universal cognitive connectome, as we have designated in our study. Future works should apply graph theory on cognitive data in other cohorts to further corroborate this idea.

Our second aim was to investigate whether this cognitive connectome differs across early-middle-age, late-middle-age, and elderly groups. Our modular analyses as well as visual inspection of the correlation matrices showed modest differences in the cognitive connectome across age groups. Specifically, the early-middle-age group showed a cognitive connectome of three modules with moderate correlations both between-modules and within-modules. The late-middle-age group showed a cognitive connectome of four modules with stronger correlations both between-modules and within-modules, especially involving executive functions. In contrast, the elderly group showed a cognitive connectome of three modules with weaker correlations, but there was a unique correlation of processing speed with other modules. These overall similarities in the cognitive connectome across age groups were also highlighted by Salthouse and Ferrer-Caja (2003), who found a similar cognitive structure when stratifying the sample by age groups. These results support the hypothesis of a stable cognitive connectome across the adulthood and older ages in healthy aging. The increase in modules from three in the early-middle-age group to four in the late-middle-age group could be interpreted as a compensatory mechanism related to de-differentiation processes during the late-middle-age (Baltes et al., 1980; Hülür et al., 2015; Gonzalez-Burgos et al., 2019). This would help to partially maintain cognitive performance during late-middle age, as observed in our data. The reduction in cognitive performance in the elderly, particularly in processing speed and procedural memory, together with the decrease in modules from four in the late-middle-age group to three in the elderly group and the prominent finding of differentiation (weak correlations among variables) further support this interpretation and suggest an aberrant organization in the elderly (Park et al., 2004; Reuter-Lorenz and Cappell, 2008; Sleimen-Malkoun et al., 2014; Gonzalez-Burgos et al., 2019, 2021).

Other observations in regard to our second aim are that in the late-middle-age group, visual and executive domains split into two separate modules. In previous studies using the current cohort, we observed that visual abilities are among the cognitive domains that are most strongly associated with age (Machado et al., 2018). In turn, executive functions showed less prominent associations with age (Machado et al., 2018), suggesting that executive functions may be important to maintain high cognitive performance during late-middle-age adulthood (Park and Reuter-Lorenz, 2009; Gonzalez-Burgos et al., 2019). This interpretation is further supported in our current study by the observation of weaker within-module correlations in the visual module, stronger within-module correlations in the executive module, and stronger between-module correlations between the executive and the visual modules.

Our third aim was to gain a deeper understanding of the network features underlying age-related differences in the cognitive connectome of early-middle-age, late-middle-age, and elderly groups. Briefly, our findings showed that the late-middle-age group was less efficient and had a decreased modularity than the early-middle-age group. The elderly group showed decreased transitivity and higher efficiency than the other age groups. The comparison between elderly and late-middle-age groups showed that the elderly group had increased modularity and decreased average strength. We interpret these findings as follows. Regarding the late-middle-age group, the decrease in modularity could be explained by the stronger between-module correlations, which support our interpretation above as a de-differentiation process during late-middle-age (Baltes et al., 1980; Hülür et al., 2015; Gonzalez-Burgos et al., 2019). However, this pattern does not seem to promote the formation of local modules, which could explain the decrease in both global and local efficiency measures (Van den Heuvel and Sporns, 2019; Bullmore and Sporns, 2021). This finding may seem contradictory at first, but we recently demonstrated that reduced efficiency is a characteristic of individuals with higher cognitive performance (Gonzalez-Burgos et al., 2021). This interpretation is further supported in our current study by the observation that increased global efficiency was related to lower cognitive performance in the elderly group. In our current study, this occurred in the context of decreased transitivity and average strength, explained by a pattern of weak or non-existent correlations across most variables. We discussed this finding above as prominent cognitive differentiation suggestive of an aberrant organization in the elderly (Gonzalez-Burgos et al., 2019, 2021). The increased modularity in the elderly may be driven by the merging of processing speed and verbal memory measures in one module, and the less de-differentiated pattern of correlations as compared with the late-middle-age group. This finding highlights the role of processing speed in the elderly (Salthouse, 1996; Schaie, 2005; Robitaille et al., 2013), as a potential compensatory mechanism on top of the prominent role of executive functions. What we observed is that elderly individuals with better processing speed managed to keep a higher performance in verbal memory. The nodal network analyses highlighted once again the important role of executive functions in late-middle-age individuals, and the role of processing speed in the elderly (Salthouse, 1996; West, 1996; Schaie, 2005; Robitaille et al., 2013).

Altogether, our current findings support two of the main theories of aging, which postulate that executive functions and processing speed drive cognitive aging (Salthouse, 1996; West, 1996). The role of the frontal lobe and its connections with other cortical and subcortical regions is the neural substrate common to these two theories of aging. While our study is on cognitive data, we could speculate that our modular organization may reflect the differentiated nature of cortical circuitry including frontal-parietal brain networks (executive—premotor module; Nowrangi et al., 2014), separate left and right medial temporal networks (verbal and visual memory modules, respectively; Squire and Bayley, 2007), and dorsolateral prefrontal networks (procedural memory module; Alexander et al., 1986). Further, this modular organization may also reflect the integrity of the brain white matter overall (processing speed module; Gunning-Dixon and Raz, 2000; Kloppenborg et al., 2014). An important contribution of our study is that we have helped to unravel the roles of executive functions and processing speed at different age groups. In particular, we demonstrated that executive functions seem to have a more prominent role during late-middle-age and elderly, whereas processing speed seems to be more relevant during the elderly.

The current study has some limitations. We performed the modular analysis with the Newman algorithm as a means to identify the cognitive connectome. The modular solution obtained with this algorithm could differ from that obtained with other methods. To minimize this problem, we also applied another popular algorithm for modular analysis: the Louvain algorithm (Blondel et al., 2008). We obtained very similar modular solutions both with Newman and Louvain algorithms, which cross-validates our findings. Furthermore, as discussed above, similar solutions have been reported using other methods such as factorial analysis and principal component analysis. This suggests that the cognitive connectome may be quite universal and does not depend on cohort, age range, cognitive tests used, or analytical method employed. Although we removed some cognitive variables from our graph analysis due to methodological reasons, the information captured in the removed variables is likely contained in variables remaining in our graph (e.g., the component reflected by the removed discrimination test from Visual Reproduction is very likely contained in other visual discrimination tests such as the Facial Recognition Test (FRT), which was retained in our graph analysis). Hence, despite methodological choices, we believe our current findings are generalizable. Future studies should test the cognitive connectome in pathological populations, and investigate whether the variables removed in this study on healthy individuals (e.g., errors) provide relevant information in such populations. Another limitation is that we analyzed cross-sectional data to investigate cognitive aging. Hence, substantiating our current results using longitudinal designs is warranted. Finally, we have demonstrated the potential of graph theory analysis on cognitive data to investigate complex associations between multiple cognitive domains and cognitive components. We interpreted our results following the principles of connectivity proposed by Van den Heuvel and Sporns (2019), which are primarily based on functional magnetic resonance imaging data. However, the field of graph theory applied to cognitive data is in its infancy, and more progress is needed to assess whether principles and interpretations need to be adjusted and new measures developed.

In conclusion, we identified a cognitive connectome that is rather stable across age in cognitively healthy individuals. Nonetheless, several differences in network features were observed, highlighting the important role of executive functions during the late-middle-age adulthood, as well as the role of processing speed during the elderly. A novelty of our study is the use of graph theory to investigate what we called the cognitive connectome. We translated the connectome concept from the neuroimaging field (Van den Heuvel and Sporns, 2019) to cognitive data, demonstrating its potential to advance our understanding of the complexity of cognitive aging. We hope that this step opens new possibilities and encourages future studies to validate and help to establish this new concept. One of the next challenges will be to integrate the well-studied brain connectome from structural and functional neuroimaging studies (Van den Heuvel and Sporns, 2019; Bullmore and Sporns, 2021), with the cognitive connectome investigated in the current study. Understanding that integration could have several important implications, both for research and clinical work in normal and pathological aging.

## Data Availability Statement

The authors of this study are willing to share the generated dataset in order to promote transparency and replicability of research, upon reasonable request from qualified researchers. Requests to access the datasets should be directed to DF, daniel.ferreira.padilla@ki.se.

## Ethics Statement

The studies involving human participants were reviewed and approved by CEIBA; local ethics committee of the University of La Laguna (Spain). The patients/participants provided their written informed consent to participate in this study.

## Author Contributions

EG-C contributed to the design of the study, organized the database, performed statistical analyses, contributed to the interpretation of the results, and wrote the first draft of the manuscript. LG-B contributed to organize the database, interpretation of the results, and revised the final version of the manuscript. JP contributed to the interpretation of the results, and revised the final version of the manuscript. JH-C contributed to the statistical analysis, and revised the final version of the manuscript. EW obtained funding, co-supervised the study, and revised the final version of the manuscript. GV contributed to the interpretation of the results, and revised the final version of the manuscript. JB contributed to the conception and design of the study, obtained funding, co-supervised the study, and revised the final version of the manuscript. DF contributed to the the conception and design of the study, wrote sections of the manuscript, contributed to the interpretation of the results, obtained funding, and supervised the study. All authors contributed to the article and approved the submitted version.

## Conflict of Interest

The authors declare that the research was conducted in the absence of any commercial or financial relationships that could be construed as a potential conflict of interest.

## Publisher’s Note

All claims expressed in this article are solely those of the authors and do not necessarily represent those of their affiliated organizations, or those of the publisher, the editors and the reviewers. Any product that may be evaluated in this article, or claim that may be made by its manufacturer, is not guaranteed or endorsed by the publisher.

## References

[B1] Alexander G. E. DeLong M. R. Strick P. L. (1986). Parallel organization of functionally segregated circuits linking basal ganglia and cortex. Annu. Rev. Neurosci. 9, 357–381. 10.1146/annurev.ne.09.030186.002041 3085570

[B2] Aribisala B. S. Royle N. A. Valdés Hernández M. C. Murray C. Penke L. Gow A.. (2014). Potential effect of skull thickening on the associations between cognition and brain atrophy in ageing. Age Ageing 43, 712–716. 10.1093/ageing/afu070 24936580

[B3] Baltes P. B. Cornelius S. W. Spiro A. Nesselroade J. R. Willis S. L. (1980). Integration versus differentiation of fluid/crytallized intelligence in old age. Dev. Psychol. 16, 625–635. 10.1037/0012-1649.16.6.625

[B4] Blessed G. Tomlinson B. E. Roth M. (1968). The association between quantitative measures of dementia and of senile change in the cerebral grey matter of elderly subjects. Br. J. Psychiatry 114, 797–811. 10.1192/bjp.114.512.797 5662937

[B5] Blondel V. D. Guillaume J. L. Lambiotte R. Lefebvre E. (2008). Fast unfolding of communities in large networks. J. Stat. Mech. 10:P10008. 10.1088/1742-5468/2008/10/p10008

[B6] Bullmore E. Sporns O. (2021). The economy of brain network organization. Nat. Rev. Neurosci. 13, 336–349. 10.1038/nrn3214 22498897

[B7] Cedres N. Machado A. Molina Y. Diaz-Galvan P. Hernández-Cabrera J. A. Barroso J.. (2019). Subjective cognitive decline below and above the age of 60: a multivariate study on neuroimaging, cognitive, clinical and demographic measures. J. Alzheimers Dis. 68, 295–309. 10.3233/JAD-180720 30741680

[B8] Chong J. S. X. Ng K. K. Tandi J. Wang C. Poh J. H. Lo J. C.. (2019). Longitudinal changes in the cerebral cortex functional organization of healthy elderly. J. Neurosci. 39, 5534–5550. 10.1523/JNEUROSCI.1451-18.2019 31109962 PMC6616287

[B9] Costa P. S. Santos N. C. Cunha P. Palha J. A. Sousa N. (2013). The use of bayesian latent class cluster models to classify patterns of cognitive performance in healthy ageing. PLoS One 8:e71940. 10.1371/journal.pone.0071940 23977183 PMC3748115

[B11] Erkinjuntti T. Hokkanen L. Sulkava R. Palo J. (1988). The Blessed Dementia Scale as a screening test for dementia. Int. J. Geriatr. Psychiatry 3, 267–273. 10.1002/gps.930030406

[B12] Fellows R. P. Schmitter-Edgecombe M. (2015). Between-domain cognitive dispersion and functional abilities in older adults. J. Clin. Exp. Neuropsychol. 37, 1013–1023. 10.1080/13803395.2015.1050360 26300441 PMC4874189

[B13] Ferreira D. Bartrés-Faz D. Nygren L. Rundkvist L. J. Molina Y. Machado A.. (2016). Different reserve proxies confer overlapping and unique endurance to cortical thinning in healthy middle-aged adults. Behav. Brain Res. 311, 375–383. 10.1016/j.bbr.2016.05.061 27263072

[B14] Ferreira D. Correia R. Nieto A. Machado A. Molina Y. Barroso J. (2015). Cognitive decline before the age of 50 can be detected with sensitive cognitive measures. Psicothema 27, 216–222. 10.7334/psicothema2014.192 26260927

[B15] Ferreira D. Machado A. Molina Y. Nieto A. Correia R. Westman E.. (2017). Cognitive variability during middle-age: possible association with neurodegeneration and cognitive reserve. Front. Aging Neurosci. 9:188. 10.3389/fnagi.2017.00188 28649200 PMC5465264

[B16] Ferreira D. Pereira J. B. Volpe G. Westman E. (2019). Subtypes of Alzheimer’s disease display distinct network abnormalities extending beyond their pattern of brain atrophy. Front. Neurol. 10:524. 10.3389/fneur.2019.00524 31191430 PMC6547836

[B17] Folstein M. F. Folstein S. E. McHugh P. R. (1975). “Mini-mental state”. A practical method for grading the cognitive state of patients for the clinician. J. Psychiatr. Res. 12, 189–198. 10.1016/0022-3956(75)90026-6 1202204

[B18] Garcia-Ramos C. Lin J. J. Kellermann T. S. Bonilha L. Prabhakaran V. Hermann B. P. (2016). Graph theory and cognition: a complementary avenue for examining neuropsychological status in epilepsy. Epilepsy Behav. 64, 329–335. 10.1016/j.yebeh.2016.02.032 27017326 PMC5035172

[B19] Garcia-Ramos C. Lin J. J. Prabhakaran V. Hermann B. P. (2015). Developmental reorganization of the cognitive network in pediatric epilepsy. PLoS One 10:e0141186. 10.1371/journal.pone.0141186 26505900 PMC4624435

[B20] Genovese C. R. Lazar N. A. Nichols T. (2002). Thresholding of statistical maps in functional neuroimaging using the false discovery rate. NeuroImage 15, 870–878. 10.1006/nimg.2001.1037 11906227

[B21] Gonzalez-Burgos L. Barroso J. Ferreira D. (2020). Cognitive reserve and network efficiency as compensatory mechanisms of the effect of aging on phonemic fluency. Aging 12, 23351–23378. 10.18632/aging.202177 33203801 PMC7746387

[B22] Gonzalez-Burgos L. Hernández-Cabrera J. A. Westman E. Barroso J. Ferreira D. (2019). Cognitive compensatory mechanisms in normal aging: a study on verbal fluency and the contribution of other cognitive functions. Aging 11, 4090–4106. 10.18632/aging.102040 31232698 PMC6628999

[B23] Gonzalez-Burgos L. Pereira J. B. Mohanty R. Barroso J. Westman E. Ferreira D. (2021). Cortical networks underpinning compensation of verbal fluency in normal aging. Cereb. Cortex 31, 3832–3845. 10.1093/cercor/bhab052 33866353 PMC8258442

[B24] Gronwall D. (1977). Paced auditory serial-addition task: a measure of recovery from concussion. Percept. Mot. Skills 44, 367–373. 10.2466/pms.1977.44.2.367 866038

[B25] Gunning-Dixon F. M. Raz N. (2000). The cognitive correlates of white matter abnormalities in normal aging: a quantitative review. Neuropsychology 14, 224–232. 10.1037//0894-4105.14.2.224 10791862

[B26] Habeck C. Steffener J. Barulli D. Gazes Y. Razlighi Q. Shaked D.. (2015). Making cognitive latent variables manifest: distinct neural networks for fluid reasoning and processing speed. J. Cogn. Neurosci. 27, 1249–1258. 10.1162/jocn_a_00778 25539045 PMC4416986

[B27] Harada C. N. Natelson Love M. C. Triebel K. L. (2013). Normal cognitive aging. Clin. Geriatr. Med. 29, 737–752. 10.1016/j.cger.2013.07.002 24094294 PMC4015335

[B28] Hayden K. M. Kuchibhatla M. Romero H. R. Plassman B. L. Burke J. R. Browndyke J. N.. (2014). Pre-clinical cognitive phenotypes for Alzheimer disease: a latent profile approach. Am. J. Geriatr. Psychiatry 22, 1364–1374. 10.1016/j.jagp.2013.07.008 24080384 PMC3968245

[B29] Heilman K. M. (1995). “Attentional asymmetries,” in Brain Asymmetry, eds Davidson R. J. Hugdahl K. (Massachussetts, MA: MIT Press), 217–234.

[B30] Hoogendam Y. Y. Hofman A. van der Geest J. N. van der Lugt A. Ikram M. A. (2014). Patterns of cognitive function in aging: the Rotterdam Study. Eur. J. Epidemiol. 29, 133–140. 10.1007/s10654-014-9885-4 24553905

[B31] Hülür G. Ram N. Willis S. L. Schaie K. W. Gerstorf D. (2015). Cognitive dedifferentiation with increasing age and proximity of death: within-person evidence from the Seattle Longitudinal Study. Psychol. Aging 30, 311–323. 10.1037/a0039260 25961879

[B32] Jonker F. Weeda W. Rauwerda K. Scherder E. (2019). The bridge between cognition and behavior in acquired brain injury: a graph theoretical approach. Brain Behav. 9:e01208. 10.1002/brb3.1208 30729721 PMC6422716

[B33] Jung J. Visser M. Binney R. J. Lambon Ralph M. A. (2018). Establishing the cognitive signature of human brain networks derived from structural and functional connectivity. Brain Struct. Funct. 223, 4023–4038. 10.1007/s00429-018-1734-x 30120553 PMC6267264

[B34] Kellermann T. S. Bonilha L. Lin J. J. Hermann B. P. (2015). Mapping the landscape of cognitive development in children with epilepsy. Cortex 66, 1–8. 10.1016/j.cortex.2015.02.001 25776901 PMC4405468

[B35] Kloppenborg R. P. Nederkoorn P. J. Geerlings M. I. van den Berg E. (2014). Presence and progression of white matter hyperintensities and cognition: a meta-analysis. Neurology 82, 2127–2138. 10.1212/WNL.0000000000000505 24814849

[B36] Kong X.-Z. Mathias S. R. Guadalupe T. ENIGMA Laterality Working Group Glahn D. C. Franke B.. (2018). Mapping cortical brain asymmetry in 17,141 healthy individuals worldwide *via* the ENIGMA Consortium. Proc. Natl. Acad. Sci. U S A 115, E5154–E5163. 10.1073/pnas.1718418115 29764998 PMC5984496

[B37] Lachman M. E. (2004). Development in midlife. Annu. Rev. Psychol. 55, 305–331. 10.1146/annurev.psych.55.090902.141521 14744218

[B38] Lee D. H. Lee P. Seo S. W. Roh J. H. Oh M. Oh J. S.. (2019). Neural substrates of cognitive reserve in Alzheimer’s disease spectrum and normal aging. NeuroImage 186, 690–702. 10.1016/j.neuroimage.2018.11.053 30503934

[B40] Machado A. Barroso J. Molina Y. Nieto A. Díaz-Flores L. Westman E.. (2018). Proposal for a hierarchical, multidimensional, and multivariate approach to investigate cognitive aging. Neurobiol. Aging 71, 179–188. 10.1016/j.neurobiolaging.2018.07.017 30149289

[B41] Mårtensson G. Pereira J. B. Mecocci P. Vellas B. Tsolaki M. Kłoszewska I.. (2018). Stability of graph theoretical measures in structural brain networks in Alzheimer’s disease. Sci. Rep. 8:11592. 10.1038/s41598-018-29927-0 30072774 PMC6072788

[B42] Mijalkov M. Kakaei E. Pereira J. B. Westman E. Volpe G. Alzheimer’s Disease Neuroimaging Initiative. (2017). BRAPH: a graph theory software for the analysis of brain connectivity. PLoS One 12:e0178798. 10.1371/journal.pone.0178798 28763447 PMC5538719

[B43] Mitchell M. B. Shaughnessy L. W. Shirk S. D. Yang F. M. Atri A. (2012). Neuropsychological test performance and cognitive reserve in healthy aging and the Alzheimer’s disease spectrum: a theoretically driven factor analysis. J. Int. Neuropsychol. Soc. 18, 1071–1080. 10.1017/S1355617712000859 23039909 PMC3600814

[B44] Mungas D. Heaton R. Tulsky D. Zelazo P. D. Slotkin J. Blitz D.. (2014). Factor structure, convergent validity, and discriminant validity of the NIH Toolbox Cognitive Health Battery (NIHTB-CHB) in adults. J. Int. Neuropsychol. Soc. 20, 579–587. 10.1017/S1355617714000307 24960474 PMC4103956

[B45] Newman M. E. (2004). Fast algorithm for detecting community structure in networks. Phys. Rev. E Stat. Nonlin. Soft. Matter Phys. 69:066133. 10.1103/PhysRevE.69.066133 15244693

[B46] Nielsen S. Wilms L. I. (2015). Cognitive aging on latent constructs for visual processing capacity: a novel structural equation modeling framework with causal assumptions based on a theory of visual attention. Front. Psychol. 5:1596. 10.3389/fpsyg.2014.01596 25642206 PMC4295434

[B47] Nowrangi M. A. Lyketsos C. Rao V. Munro C. A. (2014). Systematic review of neuroimaging correlates of executive functioning: converging evidence from different clinical populations. J. Neuropsychiatry Clin. Neurosci. 26, 114–125. 10.1176/appi.neuropsych.12070176 24763759 PMC5171230

[B48] Oh H. Madison C. Haight T. J. Markley C. Jagust W. J. (2012). Effects of age and β-amyloid on cognitive changes in normal elderly people. Neurobiol. Aging 33, 2746–2755. 10.1016/j.neurobiolaging.2012.02.008 22429886 PMC3381075

[B49] Oschwald J. Guye S. Liem F. Rast P. Willis S. Röcke C.. (2019). Brain structure and cognitive ability in healthy aging: a review on longitudinal correlated change. Rev. Neurosci. 31, 1–57. 10.1515/revneuro-2018-0096 31194693 PMC8572130

[B50] Park D. C. Polk T. A. Park R. Minear M. Savage A. Smith M. R. (2004). Aging reduces neural specialization in ventral visual cortex. Proc. Natl. Acad. Sci. U S A 101, 13091–13095. 10.1073/pnas.0405148101 15322270 PMC516469

[B51] Park D. C. Reuter-Lorenz P. (2009). The adaptive brain: aging and neurocognitive scaffolding. Annu. Rev. Psychol. 60, 173–196. 10.1146/annurev.psych.59.103006.093656 19035823 PMC3359129

[B52] Pereira J. B. Strandberg T. O. Palmqvist S. Volpe G. van Westen D. Westman E.. (2018). Amyloid network topology characterizes the progression of Alzheimer’s disease during the predementia stages. Cereb. Cortex 28, 340–349. 10.1093/cercor/bhx294 29136123 PMC6454565

[B53] Pfeffer R. I. Kurosaki T. T. Harrah C. H. Jr. Chance J. M. Filos S. (1982). Measurement of functional activities in older adults in the community. J. Gerontol. 37, 323–329. 10.1093/geronj/37.3.323 7069156

[B54] Reas E. T. Laughlin G. A. Bergstrom J. Kritz-Silverstein D. Barrett-Connor E. McEvoy L. K. (2017). Effects of sex and education on cognitive change over a 27-year period in older adults: the rancho bernardo study. Am. J. Geriatr. Psychiatry 25, 889–899. 10.1016/j.jagp.2017.03.008 28433548 PMC5522346

[B55] Reuter-Lorenz P. A. Cappell K. A. (2008). Neurocognitive aging and the compensation hypothesis. Curr. Dir. Psychol. Sci. 17, 177–182. 10.1111/j.1467-8721.2008.00570.x

[B56] Rizio A. A. Diaz M. T. (2016). Language, aging and cognition: frontal aslant tract and superior longitudinal fasciculus contribute toward working memory performance in older adults. NeuroReport 27, 689–693. 10.1097/WNR.0000000000000597 27138951 PMC4955947

[B57] Robitaille A. Piccinin A. M. Muniz-Terrera G. Hoffman L. Johansson B. Deeg D.. (2013). Longitudinal mediation of processing speed on age-related change in memory and fluid intelligence. Psychol. Aging 28, 887–901. 10.1037/a0033316 23957224 PMC4014000

[B58] Salthouse T. A. (1996). The processing-speed theory of adult age differences in cognition. Psychol. Rev. 103, 403–428. 10.1037/0033-295x.103.3.403 8759042

[B59] Salthouse T. A. (2009). When does age-related cognitive decline begin? Neurobiol. Aging 30, 507–514. 10.1016/j.neurobiolaging.2008.09.023 19231028 PMC2683339

[B60] Salthouse T. A. (2010). Selective review of cognitive aging. J. Int. Neuropsychol. Soc. 16, 754–760. 10.1017/S1355617710000706 20673381 PMC3637655

[B61] Salthouse T. A. (2016). Continuity of cognitive change across adulthood. Psychon. Bull. Rev. 23, 932–939. 10.3758/s13423-015-0910-8 26238759 PMC4740316

[B62] Salthouse T. A. Ferrer-Caja E. (2003). What needs to be explained to account for age-related effects on multiple cognitive variables? Psychol. Aging 18, 91–110. 10.1037/0882-7974.18.1.91 12641315

[B63] Salthouse T. A. Habeck C. Razlighi Q. Barulli D. Gazes Y. Stern Y. (2015). Breadth and age-dependency of relations between cortical thickness and cognition. Neurobiol. Aging 36, 3020–3028. 10.1016/j.neurobiolaging.2015.08.011 26356042 PMC4609615

[B64] Schaie K. W. (2005). What can we learn from longitudinal studies of adult development? Res. Hum. Dev. 2, 133–158. 10.1207/s15427617rhd0203_4 16467912 PMC1350981

[B65] Schroeder D. H. Salthouse T. A. (2004). Age-related effects on cognition between 20 and 50 years of age. Pers. Individual Differences 36, 393–404. 10.1016/s0191-8869(03)00104-1

[B66] Sleimen-Malkoun R. Temprado J. J. Hong S. L. (2014). Aging induced loss of complexity and dedifferentiation: consequences for coordination dynamics within and between brain, muscular and behavioral levels. Front. Aging Neurosci. 6:140. 10.3389/fnagi.2014.00140 25018731 PMC4073624

[B67] Springer J. A. Binder J. R. Hammeke T. A. Swanson S. J. Frost J. A. Bellgowan P. S.. (1999). Language dominance in neurologically normal and epilepsy subjects: a functional MRI study. Brain 122, 2033–2046. 10.1093/brain/122.11.2033 10545389

[B68] Squire L. R. Bayley P. J. (2007). The neuroscience of remote memory. Curr. Opin. Neurobiol. 17, 185–196. 10.1016/j.conb.2007.02.006 17336513 PMC2277361

[B69] Tisserand D. J. Jolles J. (2003). Special issue on the involment of prefrontal networks in cognitive ageing. Cortex 39, 1107–1128. 10.1016/S0010-9452(08)70880-3 14584569

[B70] Van den Heuvel M. P. Sporns O. (2019). A cross-disorder connectome landscape of brain dysconnectivity. Nat. Rev. Neurosci. 20, 435–446. 10.1038/s41583-019-0177-6 31127193 PMC8864539

[B71] Viroli C. (2012). Using factor mixture analysis to model heterogeneity, cognitive structure, and determinants of dementia: an application to the Aging, Demographics, and Memory Study. Stat. Med. 31, 2110–2122. 10.1002/sim.5320 22415898

[B72] Wechsler D. (1997a). Wechsler Adult Intelligence Scale—Administration and Scoring Manual. 3rd Edn. San Antonio, TX: The Psychological Corporation.

[B73] Wechsler D. (1997b). Wechsler Memory Scale—Third Edition Technical Manual. 3rd Edn. San Antonio, TX: The Psychological Corporation.

[B100] Welton T. Kent D. A. Auer D. P. Dineen R. A. (2015). Reproducibility of graph-theoretic brain network metrics: a systematic review. Brain Connect. 5, 193–202. 10.1089/brain.2014.0313 25490902 PMC4432917

[B74] West R. (2001). The transient nature of executive control processes in younger and older adults. Eur. J. Cogn. Psychol. 13, 91–105. 10.1080/09541440042000232

[B75] West R. L. (1996). An application of prefrontal cortex function theory to cognitive aging. Psychol. Bull. 120, 272–292. 10.1037/0033-2909.120.2.272 8831298

[B76] Willis S. L. Martin M. Rocke C. (2010). Longitudinal perspectives on midlife development: stability and change. Eur. J. Ageing 7, 131–134. 10.1007/s10433-010-0162-4 28798623 PMC5547357

[B77] Winblad B. Palmer K. Kivipelto M. Jelic V. Fratiglioni L. Wahlund L. O.. (2004). Mild cognitive impairment—beyond controversies, towards a consensus: report of the international working group on mild cognitive impairment. J. Intern. Med. 256, 240–246. 10.1111/j.1365-2796.2004.01380.x 15324367

[B78] Xia Y. Chen Q. Shi L. Li M. Z. Gong W. Chen H.. (2019). Tracking the dynamic functional connectivity structure of the human brain across the adult lifespan. Hum. Brain Mapp. 40, 717–728. 10.1002/hbm.24385 30515914 PMC6865727

[B79] Zaidel D. W. (1990). “Memory and spatial cognition following commissurotomy,” in Handbook of Neuropsychology, vol 4, eds Boller F. Grafman J. (Amsterdam: Elsevier).

[B80] Zhu W. Wen W. He Y. Xia A. Anstey K. J. Sachdev P. (2012). Changing topological patterns in normal aging using large-scale structural networks. Neurobiol. Aging 33, 899–913. 10.1016/j.neurobiolaging.2010.06.022 20724031

