# Supplementary material for: The Cognitive Connectome in Healthy Aging
Source: Front Aging Neurosci. 2021 Aug 18;13:694254. doi: 10.3389/fnagi.2021.694254 (PMC8416612; doi:10.3389/fnagi.2021.694254)
Supplement: Supplementary file 1 [file Data_Sheet_1.docx]

**Supplementary figures and table**


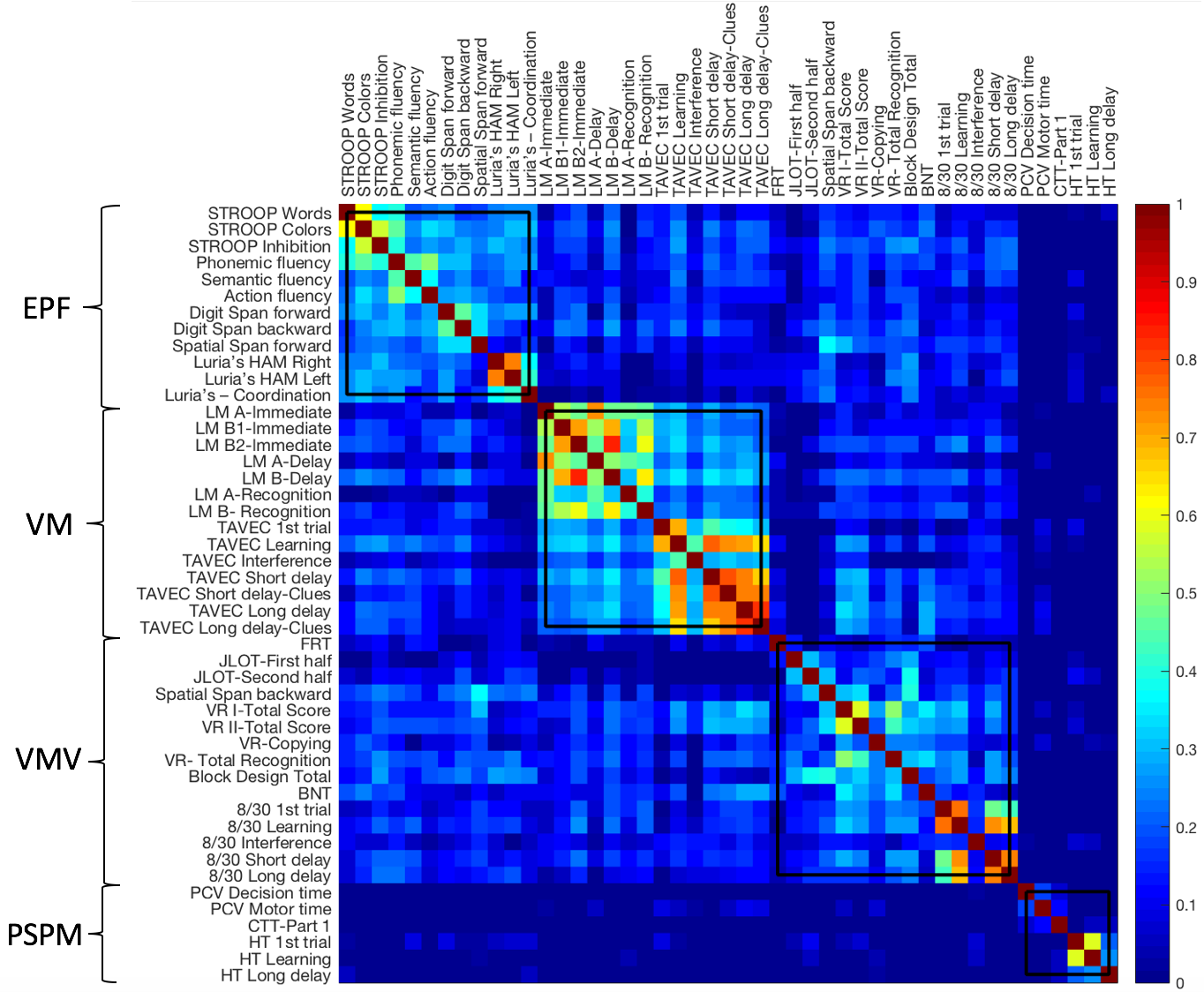


**Supplementary Figure 1:** Weighted correlation matrix of whole cohort (N = 334) sorted out by cognitive modules obtained with the Louvain algorithm. Pearson’s correlation coefficients were used to build the matrix. The colour bar indicates the strength of the Pearson’s correlation coefficients: colder colours represent weaker correlations, while warmer colours represent stronger correlations. EPF, Executive Premotor Functions; VM, Verbal Memory; VMV, Visual Memory Visuospatial; PSPM, Processing Speed Procedural Memory; LM, Logical Memory; FRT, Facial Recognition Test; JLOT, Judgment of Line Orientation Test; VR, Visual Reproduction; BNT, Boston Naming Test; HT, Hanoi Tower; PCV, PC-Vienna System; CTT, Color Trails Test.


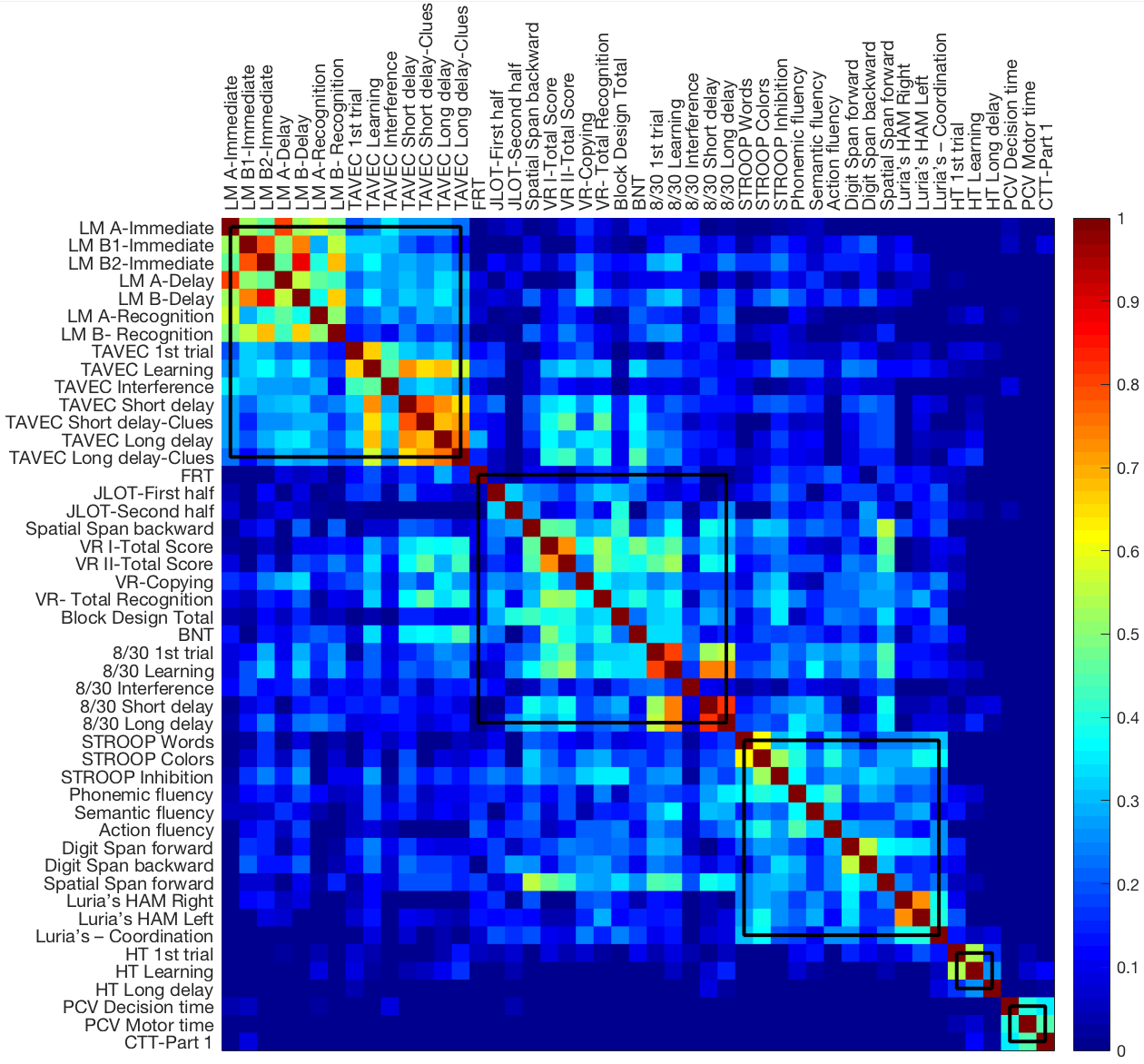


**Supplementary Figure 2:** Correlation matrix of the early-middle-age group, keeping the five modules of the whole cohort fixed. Newman algorithm was used for modular analysis. Pearson’s correlation coefficients were used to build the matrix. The colour bar indicates the strength of the Pearson’s correlation coefficients: colder colours represent weaker correlations, while warmer colours represent stronger correlations. LM, Logical Memory; FRT, Facial Recognition Test; JLOT, Judgment of Line Orientation Test; VR, Visual Reproduction; BNT, Boston Naming Test; HT, Hanoi Tower; PCV, PC-Vienna System; CTT, Color Trails Test.


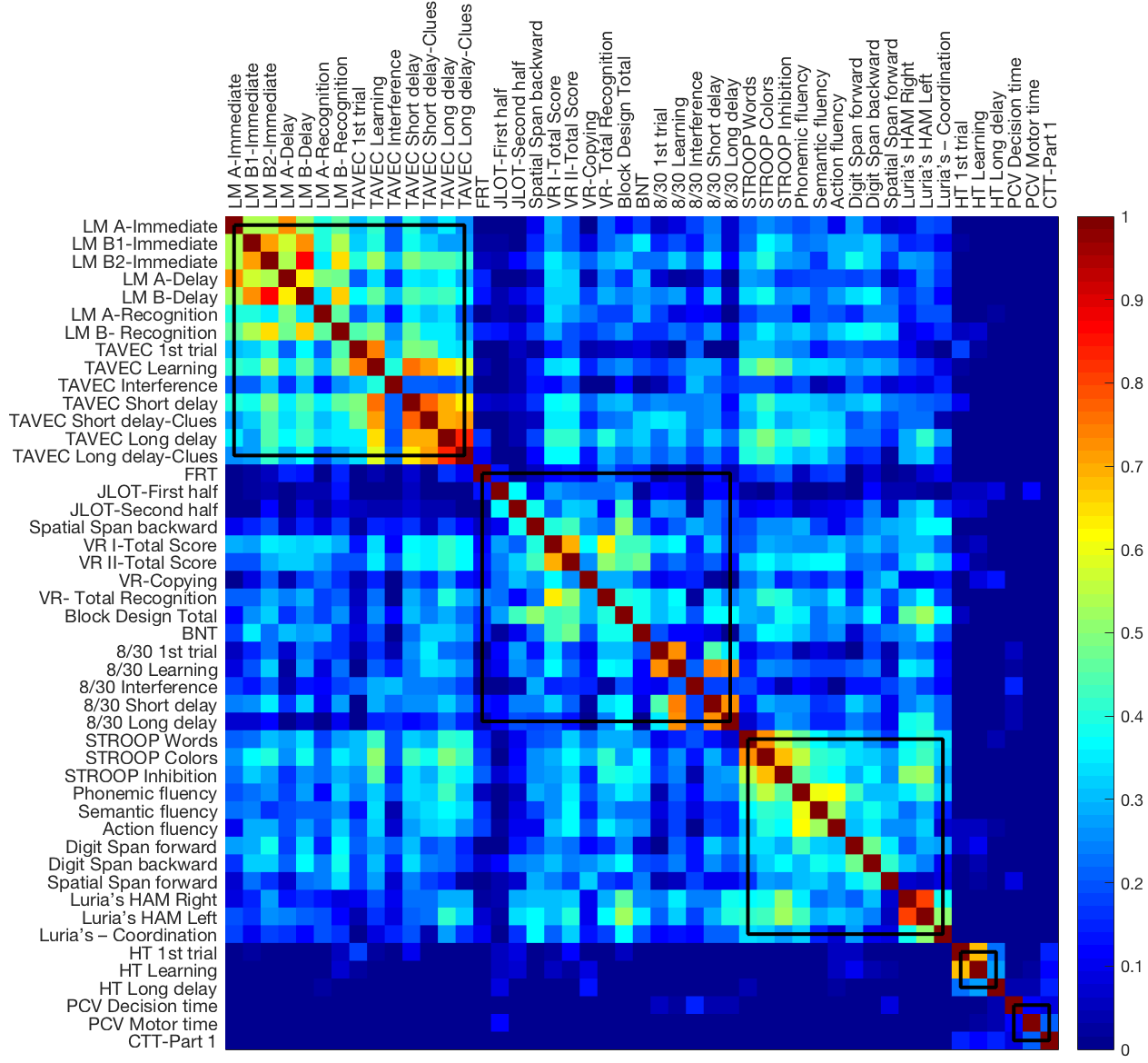


**Supplementary Figure 3:** Correlation matrix of the late-middle-age group, keeping the five modules of the whole cohort fixed. Newman algorithm was used for modular analysis. Pearson’s correlation coefficients were used to build the matrix. The colour bar indicates the strength of the Pearson’s correlation coefficients: colder colours represent weaker correlations, while warmer colours represent stronger correlations. LM, Logical Memory; FRT, Facial Recognition Test; JLOT, Judgment of Line Orientation Test; VR, Visual Reproduction; BNT, Boston Naming Test; HT, Hanoi Tower; PCV, PC-Vienna System; CTT, Color Trails Test.


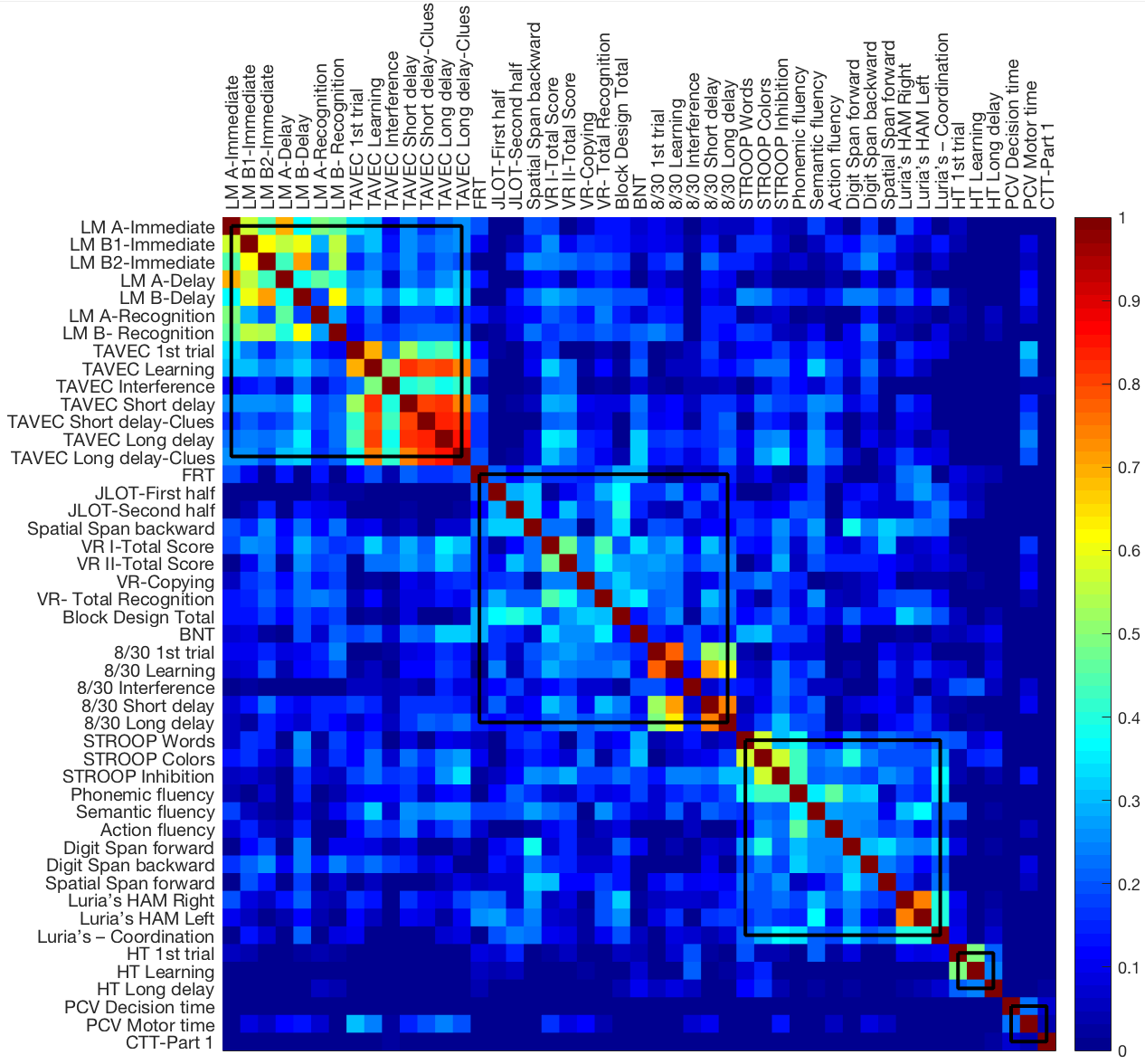


**Supplementary Figure 4:** Correlation matrix of the elderly group, keeping the five modules of the whole cohort fixed. Newman algorithm was used for modular analysis. Pearson’s correlation coefficients were used to build the matrix. The colour bar indicates the strength of the Pearson’s correlation coefficients: colder colours represent weaker correlations, while warmer colours represent stronger correlations. LM, Logical Memory; FRT, Facial Recognition Test; JLOT, Judgment of Line Orientation Test; VR, Visual Reproduction; BNT, Boston Naming Test; HT, Hanoi Tower; PCV, PC-Vienna System; CTT, Color Trails Test.

**
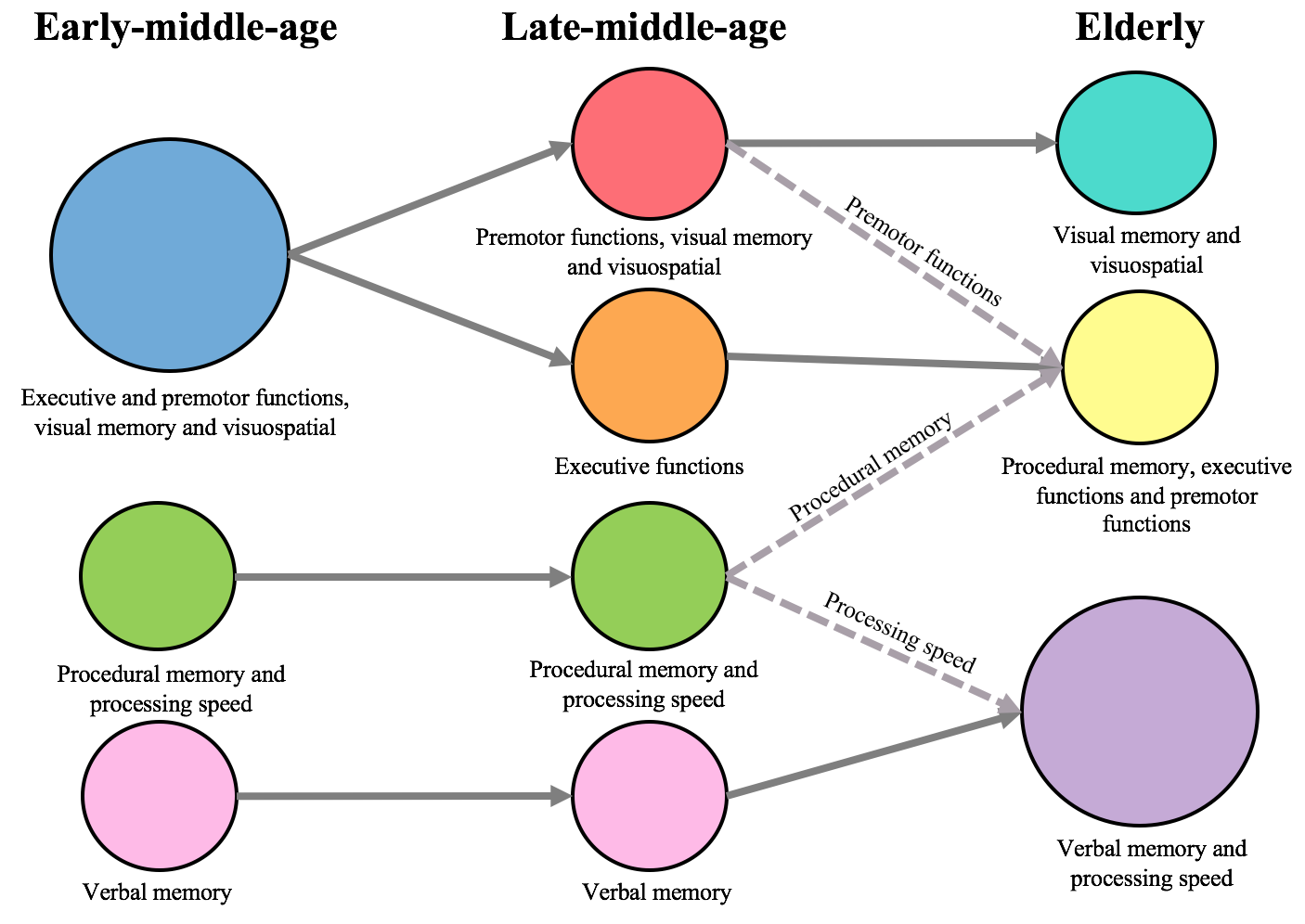
**

**Supplementary Figure 5:** Schematic representation of cognitive modules using the Newman algorithm. Cognitive modules were obtained using the Newman algorithm from the set of 47 cognitive variables, previously controlled for crystalized intelligence (WAIS-III Information subtest).


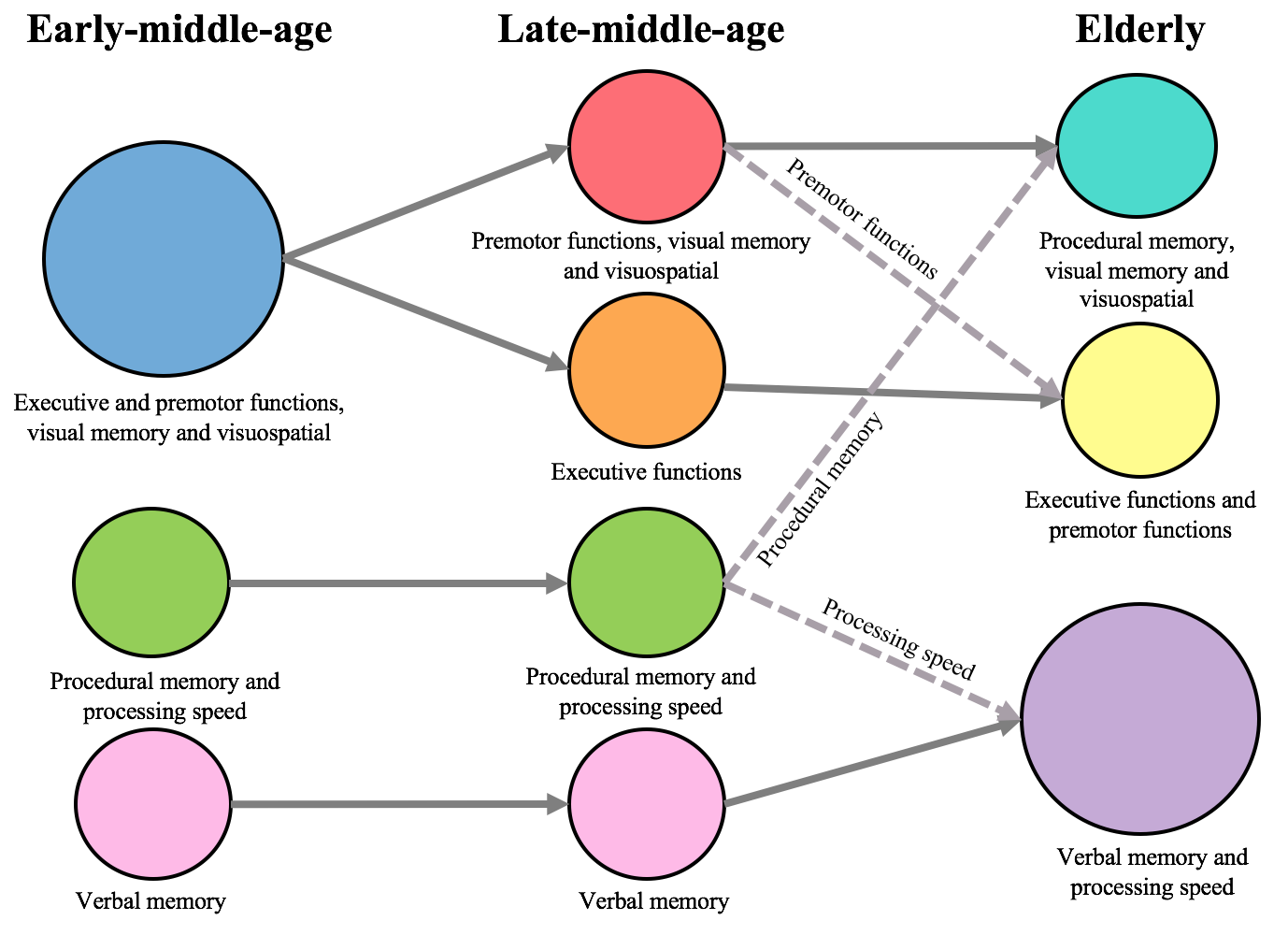


**Supplementary Figure 6:** Schematic representation of cognitive modules using the Louvain algorithm. Cognitive modules were obtained using the Louvain algorithm in each age group separately, when reducing the set of 47 cognitive variables previously controlled for crystalized intelligence (WAIS-III Information subtest).

**Table 1. Neuropsychological v cognitive assessment**

| **Neuropsychological Test; Reference** | **Cognitive measures** | **Most prominent cognitive component** |
| --- | --- | --- |
| PC-Vienna System (PCV); Schuhfried (1992) | PCV Decision time | Cognitive reaction time |
|  | PCV Motor time | Motor reaction time |
| Stroop Test (STROOP); Golden (1978) | STROOP Words | Processing speed |
|  | STROOP Colors | Processing speed |
|  | STROOP Inhibition | Cognitive inhibition (Executive function) |
| Color Trails Test (CTT); D’Elia and Satz (1989) | CTT-Part 1 | Focusing/visual tracking |
| Verbal fluency; Benton, Hamsher & Sivan (1989) (letters and animals); Piatt et al. (1999) (actions) | Phonemic fluency | Phonemic (letters), semantic (animals) and actions. Executive function |
|  | Semantic fluency |  |
|  | Action fluency |  |
| Facial Recognition Test (FRT); Benton et al. (1983) | FRT | Visuoperceptive abilities |
| Judgment of Line Orientation Test (JLOT); Benton et al. (1983) | JLOT-First half | Visuospatial abilities |
|  | JLOT-Second half |  |
| Digit Span; Wechsler (1997b) | Digit Span forward | Working memory: amplitude (forward) |
|  | Digit Span backward | Working memory: manipulation (backward) |
| Spatial Span; Wechsler (1997b) | Spatial Span forward | Working memory: amplitude (forward) |
|  | Spatial Span backward | Working memory: manipulation (backward) |
| Logical Memory (LM); Wechsler (1997b) | LM A-Immediate | Encoding (Verbal memory) |
|  | LM B1-Immediate |  |
|  | LM B2-Immediate |  |
|  | LM A-Delay | Retrieval (Verbal memory) |
|  | LM B-Delay |  |
|  | LM A-Recognition | Storage (Verbal memory) |
|  | LM B- Recognition |  |
| *Test de Aprendizaje Verbal España-Complutense* (TAVEC, Spanish Version of California Verbal Learning Test); Benedet & Alejandre (1998) | TAVEC 1^st^ trial | Encoding (Verbal memory) |
|  | TAVEC Learning |  |
|  | TAVEC Interference | Proactive interference in encoding |
|  | TAVEC Short delay | Retrieval (Verbal memory) |
|  | TAVEC Short delay-Clues |  |
|  | TAVEC Long delay |  |
|  | TAVEC Long delay-Clues |  |
| Visual Reproduction (VR); Wechsler (1997b) | VR I-Total Score | Encoding (Visual memory) |
|  | VR II-Total Score | Retrieval (Visual memory) |
|  | VR-Copying | 2-D visuoconstructive abilities |
|  | VR- Total Recognition | Storage (Visual memory) |
| Luria’s Premotor Functions (Luria’s) | Luria’s HAM Right | Hand alternative movements, |
|  | Luria’s HAM Left |  |
|  | Luria’s – Coordination | Motor coordination |
| Block Design; Wechsler (1997a) | Block Design Total | 3-D visuoconstructive abilities |
| Boston Naming Test (BNT); Kaplan et al. (1983) | BNT | Lexical access by visual confrontation |
| 8/30 Spatial Recall Test (8/30 SRT); Modification of the 7/24 SRT: Rao et al. (1984) | 8/30 1^st^ trial | Encoding (Visuospatial memory) |
|  | 8/30 Learning |  |
|  | 8/30 Interference | Proactive interference in encoding |
|  | 8/30 Short delay | Retrieval (Visuospatial memory) |
|  | 8/30 Long delay |  |
| Hanoi Tower (HT); Simon (1975) | HT 1^st^ trial | Planning, flexibility and problem resolution (executive function) and motor learning (procedural memory) |
|  | HT Learning |  |
|  | HT Long delay |  |

The table describes neuropsychological tests, references and main cognitive component related with each cognitive measure according to Lezak et al. (2012). The second column in the table shows the list of cognitive measures included as nodes to construct the cognitive connectome.
